# Antibiotic Prophylaxis for Cesarean Delivery: A Survey of Anesthesiologists

**DOI:** 10.1155/2020/3741608

**Published:** 2020-12-16

**Authors:** Emily S. Reiff, Ashraf S. Habib, Brendan Carvalho, Karthik Raghunathan

**Affiliations:** ^1^Durham, NC, Department of Obstetrics and Gynecology, Duke University, USA; ^2^Durham, NC, Department of Anesthesiology, Duke University, USA; ^3^Palo Alto, CA, Department of Anesthesiology, Perioperative and Pain Medicine, Stanford University, USA

## Abstract

**Background:**

The most common complication after cesarean delivery is surgical site infection. Antibiotic prophylaxis reduces infectious morbidity and current anesthetic quality metrics include preincision antibiotic prophylaxis. Recently, studies suggest reductions in infectious morbidity with the addition of azithromycin for unscheduled cesarean delivery. Larger doses of cefazolin are recommended for morbidly obese women, but evidence is conflicting. The aim of this study was to survey anesthesiologists to assess current practice for antibiotic prophylaxis for cesarean delivery.

**Methods:**

We invited a random sample of 10,000 current members of the American Society of Anesthesiologists to complete an online survey about their current practice of antibiotic prophylaxis for cesarean delivery in November 2017. The survey included questions similar to a previous survey on this topic in 2012.

**Results:**

The response rate was 12.2% (*n* = 1223). Most respondents had at least 15 years of experience (684, 55.9%), work at a nonteaching or community hospital (729, 59.6%), with >500 cesarean deliveries annually (619, 50.6%), and administer obstetric anesthesia several times a week (690, 56.4%). Routine preincision antibiotic prophylaxis was reported by 1162 (95.0%) of the 1223 respondents, a substantial improvement versus the 63.5% reported in the previous study in 2012. For intrapartum cesarean deliveries, 141 (11.5%) administer azithromycin for unscheduled cesarean deliveries. Those who use cefazolin, 509 (42.5%) administer 3 g for morbidly obese women.

**Conclusion:**

Adherence to preincision antibiotic prophylaxis for cesarean delivery is very high, a significant improvement within 5 years. A minority of anesthesiologists utilize azithromycin for intrapartum cesarean deliveries. The dose of cefazolin for morbidly obese women varies widely.

## 1. Introduction

Cesarean delivery is a major risk factor for postpartum infection with a five to twenty times greater incidence as compared to vaginal delivery [1]. Prophylactic antibiotics at the time of scheduled cesarean delivery have been shown to reduce the risk of wound infection (RR 0.62, 95% CI 0.47–0.82) and endometritis (RR 0.38, 95% CI 0.24–0.61) compared to placebo or no treatment [1]. Preincision antibiotic prophylaxis also reduces an infectious composite outcome that includes maternal sepsis, wound dehiscence or evisceration, necrotizing fasciitis, pelvic abscess, and septic pelvic thrombophlebitis [2, 3]. Current American College of Obstetricians and Gynecologists (ACOG) recommendations include antibiotic prophylaxis given to all women undergoing scheduled cesarean delivery within 60 minutes prior to incision [4]. In women without significant allergies, a single dose of a first-generation cephalosporin is recommended due to its efficacy, narrow spectrum of activity, and low cost [5]. Alternatively, in women with type 1 hypersensitivity to penicillins or cephalosporins, clindamycin plus an aminoglycoside is recommended [4].

In 2012, a nationwide survey was administered to members of the American Society of Anesthesiologists regarding current antibiotic practices and found striking variability with only 63.5% reporting use of preincision prophylaxis prior to cesarean delivery [6]. However, since this survey, there have been further data and recommendations outlining the importance of preincision compared to postdelivery antibiotics in obstetric patients [7], and timely administration for all scheduled cesarean deliveries with an appropriately chosen antibiotic has become a quality metric for hospitals [8].

This survey was undertaken to examine current practices of anesthesiologists regarding antibiotic prophylaxis for cesarean delivery. The aim of the study was to evaluate if there has been a change in practice and increased adoption of the ACOG recommendations to administer preincision antibiotics in all cesarean deliveries. Additionally, we assessed the adoption rates of newer antibiotic practices including azithromycin for intrapartum cesarean deliveries and dose modification in obese parturients, as well as factors that were associated with greater integration of such practices.

## 2. Materials and Methods

The survey was reviewed and approved by the Institutional Review Board at Duke University Hospital in July 2017 and conducted by investigators at both Duke University and Stanford University. The original survey administered in 2012, which underwent internal face validity during its initial development, was utilized in this study with the addition of questions regarding adjunctive azithromycin use and cefazolin dosing in morbidly obese women (see the Appendix A in Supplementary Materials). The survey was then sent electronically by the ASA administration to 10,000 random current members of the ASA, pulled from the current membership directory in November 2017. Two weeks after the initial e-mail was sent out, a reminder e-mail was sent to the same cohort to complete the survey. The survey was then taken offline two weeks following the reminder e-mail. The primary outcome of interest was adherence to the ACOG recommendation of administration of preincision antibiotics for cesarean deliveries. Secondary outcomes included adoption of ACOG suggested practices, including azithromycin administration for intrapartum cesarean deliveries and 3 g of cefazolin for morbidly obese women. Rate of preincision antibiotic use was then compared to the prior rate obtained in the 2012 study. We also examined factors associated with adherence and integration of these antibiotic practices.

### 2.1. Statistical Analysis

Survey response data are presented as numbers (percentage). Means with standard deviations and proportions with 95% confidence intervals were calculated for summary analyses. The primary outcome of preincision antibiotic prophylaxis was compared to the prior rate in the 2012 survey using a Chi-squared test. The primary and secondary outcomes were then examined as a function of various characteristics using chi-squared tests or Fisher's exact test, where appropriate, for categorical variables, with *P* < 0.05 considered statistically significant. SAS Enterprise Guide 9.4 (©2018, Cary, NC) was used for analysis.

## 3. Results

Of the 10,000 members to which a survey was sent, 1351 (13.5%) returned the survey with 1223 (12.2%) returning a complete response, 25 (0.3%) returning an incomplete response, and 103 (1.0%) stating they did not currently perform obstetric anesthesia. Most respondents had at least 15 years of experience (684, 55.9%), work at a nonteaching or community hospital (729, 59.6%) with >500 cesarean deliveries annually (619, 50.6%), and work in obstetric anesthesia several times a week (690, 56.4%) (Table 1).

Routine preincision antibiotic prophylaxis was reported by 1162 (95.0%) respondents. For intrapartum cesarean deliveries, 141 (11.5%) administer adjunctive azithromycin. Of those who use cefazolin for prophylaxis, 509 (42.5%) administer 3 g for morbidly obese women, as compared to 654 (54.5%) who use 2 g and 36 (3%) who use 1 g (Table 2).

Routine preincision antibiotic prophylaxis rates were significantly improved from the 63.5% reported in the previous survey in 2012 (Χ^2^ (1) = 356.4, *P* < 0.0001).

Routine preincision antibiotic administration was significantly associated with the frequency a provider administers obstetric anesthesia, region in the United States of provider practice, years the provider was in practice, and annual number of cesarean deliveries at their place of employment (Table 3). Adjunctive azithromycin administration for intrapartum cesarean delivery was significantly associated with region in the United States of the provider's practice, years the provider was in practice, and annual number of cesarean deliveries at their place of employment (Table 3). Administration of 3 g of cefazolin in morbidly obese women was also significantly associated with region, years in practice, and institutional annual number of cesarean deliveries (Table 3).

## 4. Discussion

An overwhelming majority of practicing anesthesiologists administer preincision antibiotics for cesarean deliveries. This is a stark improvement from the same survey performed 5 years earlier. During those five years, more evidence surfaced demonstrating significant reduction in postoperative infectious morbidity with preincision compared to postdelivery antibiotic administration, as well as the introduction of adherence to this policy as a quality metric in anesthesia [7, 8]. There is significant variability in practice patterns across provider volume and experience, institutional volume, and region of the United States. Providers with a higher frequency of obstetric anesthesia administration and less years in practice, and institutions with more annual deliveries were more likely to administer preincision antibiotic prophylaxis. The Southwest has the highest rates of administering preincision antibiotic prophylaxis.

Since the C-SOAP trial was published in 2016 demonstrating a reduction in postoperative infectious complications for women undergoing intrapartum cesarean deliveries who receive adjunctive azithromycin, ACOG stated in September 2018 that adjunctive azithromycin can be considered by providers in women undergoing unscheduled cesarean delivery [4, 9]. Our survey, performed prior to the ACOG update in November 2017, demonstrates that a minority of anesthesiologists administered adjunctive azithromycin for unscheduled cesarean deliveries prior to the practice bulletin change. There is significant variability in practice patterns across provider experience, institutional volume, and region of the United States. Providers with less years in practice and institutions with more annual deliveries were more likely to administer adjunctive azithromycin. The Midwest had the highest rates of adjunctive azithromycin use.

Postoperative infectious morbidity is higher in morbidly obese women as compared to women with a normal BMI, up to 3- to 5-fold [10, 11]. Potential reasons for higher infectious complication in obesity include decreased tissue perfusion, higher diabetes/glucose levels, and lower relative concentration of antibiotics in morbidly obese women [12, 13]. Because of these findings, it has been postulated that an increased dose of cefazolin for antibiotic prophylaxis in morbidly obese women could increase tissue concentration and subsequently decrease postoperative infection [14]. ACOG updated their practice bulletin in September 2018 to suggest a dose of 3 g for women greater than 120 kg [4]. In our study, conducted prior to the ACOG practice bulletin update in November 2017, we found that a minority of providers use 3 g in morbidly obese women. There is significant variability in practice patterns across provider experience, institutional volume, and region of the United States. Providers with less years in practice and institutions with more annual deliveries were more likely to administer 3 g of cefazolin in morbidly obese women. The Midwest had the highest rates of use.

Given the methodology of this study, there are several inherent limitations. Surveys have intrinsic selection bias, particularly nonresponse bias in that responders may be systematically different than nonresponders. This may overestimate or underestimate the measures evaluated in this study and therefore ultimately interfere with validity of the study. Additionally, the true denominator of surveyed ASA members is unclear, as we do not know the number of providers who either did not see nor have the ability to access the survey link prior to closure. These limitations are inherent to electronic survey methodology and unavoidable in this study. Additionally, we relied on the honesty of survey respondents to answer the questions truthfully and were unable to validate their response against medical administration records. The response rate was also relatively low but consistent with similar medical e-mail surveys.

Quality metrics in anesthesia are developed to ensure patients receive the highest quality anesthesia care, provide benchmarks to track over time, and measure how practice patterns influence outcomes. Preincision antibiotic administration has commonly been included in anesthesia quality metrics as a process measure [8, 15]. The current high rate of preincision antibiotic administration calls into question whether it still remains a valid and useful quality metric for obstetric anesthesia. We postulate that a 95% benchmark is a successful measure. Given the emergent nature of some cesarean deliveries with higher clinical priorities which require quick attention, 100% adherence may not be feasible or clinically possible. Further research is required to understand the specific role of quality metrics and their relationship with quality care in obstetric anesthesia.

## 5. Conclusions

In summary, adherence to preincision antibiotic prophylaxis for cesarean delivery is very high, which is a significant improvement from the same survey conducted 5 years earlier, highlighting the importance of serial surveys over time to assess trends and guideline/information dissemination. This data challenges the use of preincision antibiotic adherence as a discriminator of anesthesia quality and suggests the need to think critically about current and proposed metrics. Additionally, a minority of anesthesiologists have implemented adjunctive azithromycin for intrapartum cesarean deliveries and 3 g of cefazolin for prophylaxis in morbidly obese women. It will be important to continue to track these process measures over time and correlate with obstetric outcomes as we continue to perform quality improvement work in an effort to reduce maternal morbidity .

## Figures and Tables

**Table 1:** Baseline characteristics of survey respondents.

Respondent and hospital characteristics	*N*	%
Anesthesiologist experience		
Currently training	4	0.3
<5 years	107	8.8
5–14 years	427	34.9
15+ years	684	55.9
Region of practice in the United States		
Northeast	248	20.3
Southeast	233	19.1
Midwest	245	20.0
Southwest	118	9.7
West	241	19.7
International	138	11.3
Hospital type (respondents could choose >1)		
University or teaching hospital	471	38.5
Nonteaching or community hospital	729	59.6
Public/military	53	4.3
Variable	14	1.1
Number of institutional cesarean deliveries annually		
<100	81	6.6
100–500	523	42.8
>500	619	50.6
Frequency working in obstetric anesthesia		
Very often (several times a week)	690	56.4
Occasionally (a few times a month)	475	38.8
Rarely (less than once a month)	38	3.1
Very rarely (only a few times a year)	20	1.6
Temporal practice change		
This is how I was trained	396	32.4
My practice changed >7 years ago	301	24.6
My practice changed 1–7 years ago	463	37.96
My practice changed within the past year	63	5.2

**Table 2:** Current antibiotic practices by anesthesiologists for cesarean delivery.

Current antibiotic practice	*N*	%
Routine prophylaxis for unlabored cesarean delivery		
Cefazolin	1187	97.1
Azithromycin	49	4.0
Ampicillin	21	1.7
Gentamicin	20	1.6
Other	49	4.0
Timing of antibiotic		
30–60 min preincision	75	6.1
15–30 min preincision	367	30.0
0–15 min preincision	720	58.9
Total preincision	1162	95.0
Postcord clamp	33	2.1
Per obstetrician	24	2.0
Not in scope of practice	4	0.3
Routine prophylaxis for labored cesarean delivery		
Cefazolin	1148	93.9
Azithromycin	141	11.5
Ampicillin	35	2.9
Gentamicin	56	4.6
Other	61	5.0
Cefazolin dose for morbidly obese women		
1 g	36	2.9
2 g	654	53.5
3 g	509	41.6
I don't routinely use cefazolin	23	1.9

**Table 3:** Bivariate analysis of preincision antibiotic prophylaxis, adjunctive azithromycin for unscheduled cesarean delivery, and 3 g cefazolin in morbidly obese women.

Characteristic	Preincision prophylaxis (%)	*P* value	Azithromycin use in intrapartum cesarean delivery (%)	*P* value	3 g cefazolin in morbidly obese women (%)	*P* value
Administration frequency of obstetric anesthesia		<0.0001		0.2286		0.6520
Very rarely (only a few times a year)	15/20 (75.0%)		1/20 (5.0%)		10/20 (50.0%)	
Rarely (less than once a month)	31/38 (81.6%)		3/38 (7.9%)		13/38 (34.2%)	
Occasionally (a few times a month)	453/475 (95.4%)		46/475 (9.7%)		202/475 (42.5%)	
Very often (several times a week)	663/690 (96.1%)		91/690 (13.2%)		284/690 (41.2%)	

Region in the United States		<0.0001		<0.0001		<0.0001
Northeast	240/248 (96.8%)		27/248 (10.9%)		97/248 (39.1%)	
Southeast	219/233 (94.0%)		20/233 (8.6%)		86/233 (36.9%)	
Midwest	238/245 (97.1%)		41/245 (16.7%)		123/244 (50.4%)	
Southwest	116/118 (98.3%)		18/118 (15.3%)		57/118 (48.3%)	
West	235/241 (97.5%)		32/241 (13.3%)		117/241 (48.6%)	
Other	114/138 (82.6%)		3/138 (2.2%)		29/138 (21.0%)	

Years in practice		0.0025		<0.0001		<0.0001
Currently training	4/4 (100.0%)		2/4 (50.0%)		3/4 (75.0%)	
<5 years	106/107 (99.1%)		26/107 (24.3%)		68/107 (63.6%)	
5–15 years	450/464 (97.0%)		68/464 (14.7%)		217/464 (46.8%)	
More than 15 years	601/647 (92.9%)		45/647 (7.0%)		221/647 (34.2%)	

Institutional annual number of cesarean deliveries		0.0043		<0.0001		0.0041
<100	70/81 (86.4%)		2/81 (2.5%)		24/81 (29.6%)	
100–500	499/523 (95.4%)		45/523 (8.6%)		202/523 (38.6%)	
>500	593/619 (95.8%)		94/619 (15.2%)		283/619 (45.7%)	

## Data Availability

The data used to support the findings of this study are available from the corresponding author upon request.

## Supplementary Materials

Supplementary Materials Online Survey.
