# Supplementary material for: Antibiotic Prophylaxis for Cesarean Delivery: A Survey of Anesthesiologists
Source: Anesthesiol Res Pract. 2020 Dec 16;2020:3741608. doi: 10.1155/2020/3741608 (PMC7787788; doi:10.1155/2020/3741608)
Supplement: Supplementary Materials — Online Survey. [file 3741608.f1.docx]

Appendix A. Perioperative Antibiotics for Cesarean Section: A Practice Survey

You are being asked to participate in this research project because you are a practicing anesthesiologist and an active member of the American Society of Anesthesiologists. As perioperative physicians, anesthesiologists play a critical role in the reduction of surgical site infections. The aims of this study are to assess current practice patterns in routine antibiotic prophylaxis for cesarean sections, and to identify factors related to variations which might help target future educational opportunities.

This study is being conducted by Ashraf Habib, MD, an anesthesiologist at Duke University and is being sent to 10,000 randomly chosen active member anesthesiologists.

Your participation is completely voluntary and you may quit at any time. You may also skip any question, but continue to complete the rest of the survey. Your responses will be kept anonymous. When research results are reported, responses will be aggregated and described in the summary. This survey should take approximately 5 minutes to complete.

For additional information, please contact Duke University Institutional Review Board at (919) 668-5111.

Thank you for participating in this effort to improve our knowledge of perioperative practices.

***Provider Demographics***

**How often do you administer spinal, epidural and/or general anesthesia for cesarean sections?**

Very often (several times a week)

Occasionally (a few times a month)

Rarely (less than once a month)

Very rarely (only a few times a year)

Never

**Please choose the state where you work now.**

**Years in practice since completion of training.**

***Hospital Characteristics***

**What best describes your primary practice setting? (select all that apply)**

University or teaching hospital (with physician trainees present)

Non-teaching or community hospital (no trainees present)

Public or military hospital (funded by city, state or federal government)

Locum with variable practice setting

**Approximately how many cesarean sections per year are performed at your primary practice institution?**

Less than 100

Between 100 and 500

More than 500

***Antibiotic Administration***

**When do you routinely administer antibiotic prophylaxis for scheduled cesarean sections?**

>30-60 minutes pre-incision

>15-30 minutes pre-incision

0-15 minutes pre-incision

Post-cord clamp (following delivery after the umbilical cord is clamped)

It varies/depends on the obstetrician

Administration of antibiotics is not within my scope of practice

**Has your practice of antibiotic administration for cesarean deliveries changed recently?**

This is how I was trained.

My practice changed more than 7 years ago.

My practice changed within the past 1-7 years.

My practice changed within the past year.

**Does your practice for urgent or emergent cesarean sections differ from that of routine cesarean sections?**

No

Yes

**What antibiotic(s) do you most commonly use for routine surgical prophylaxis for unlabored cesarean sections, if no drug allergies? Check all that apply.**

Cefazolin

Azithromycin

Ampicillin

Gentamicin

Other

**What antibiotic(s) do you most commonly use for routine surgical prophylaxis for labored cesarean sections, if no drug allergies? Check all that apply**

Cefazolin

Azithromycin

Ampicillin

Gentamicin

Other

**What dose of cefazolin do you routinely use for morbidly obese patients?**

1g

2g

3g

I do not routinely use cefazolin

**Do you and/or the obstetric team ever administer prophylactic oral antibiotics postoperatively?**

Yes

No

I don't know

**Please enter any comments you may have on whether timely perioperative antibiotic prophylaxis is appropriate as a measure of anesthesia quality:**

**Thank you for participating in this effort to improve our knowledge of perioperative practices. Once again, please be assured that your responses are completely anonymous.**

**For additional information, please contact the Duke University Institutional Review Board at (919) 668-5111.**
